# Association of Socioeconomic Status and Brain Injury With Neurodevelopmental Outcomes of Very Preterm Children

**DOI:** 10.1001/jamanetworkopen.2019.2914

**Published:** 2019-05-03

**Authors:** Isabel Benavente-Fernández, Anne Synnes, Ruth E. Grunau, Vann Chau, Chantel Ramraj, Torin Glass, Dalit Cayam-Rand, Arjumand Siddiqi, Steven P. Miller

**Affiliations:** 1 Department of Pediatrics (Neurology), The Hospital for Sick Children, Toronto, Ontario, Canada; 2 Department of Pediatrics (Neurology), University of Toronto, Toronto, Ontario, Canada; 3 Department of Pediatrics (Neonatology), University Hospital Puerta del Mar, Cadiz, Spain; 4 British Columbia Children's Hospital Research Institute, Vancouver, British Columbia, Canada; 5 Department of Pediatrics (Neonatology), University of British Columbia, British Columbia Women's Hospital and Health Centre, Vancouver, British Columbia, Canada; 6 Division of Epidemiology, Dalla Lana School of Public Health, University of Toronto, Toronto, Ontario, Canada

## Abstract

**Importance:**

Studies of socioeconomic status and neurodevelopmental outcome in very preterm neonates have not sensitively accounted for brain injury.

**Objective:**

To determine the association of brain injury and maternal education with motor and cognitive outcomes at age 4.5 years in very preterm neonates.

**Design, Setting, and Participants:**

Prospective cohort study of preterm neonates (24-32 weeks’ gestation) recruited August 16, 2006, to September 9, 2013, at British Columbia Women's Hospital in Vancouver, Canada. Analysis of 4.5-year outcome was performed in 2018.

**Main Outcomes and Measures:**

At age 4.5 years, full-scale IQ assessed using the Wechsler Primary and Preschool Scale of Intelligence, Fourth Edition, and motor outcome by the percentile score on the Movement Assessment Battery for Children, Second Edition.

**Results:**

Of 226 survivors, neurodevelopmental outcome was assessed in 170 (80 [47.1%] female). Based on the best model to assess full-scale IQ accounting for gestational age, standardized β coefficients demonstrated the effect size of maternal education (standardized β = 0.21) was similar to that of white matter injury volume (standardized β = 0.23) and intraventricular hemorrhage (standardized β = 0.23). The observed and predicted cognitive scores in preterm children born to mothers with postgraduate education did not differ in those with and without brain injury. The best-performing model to assess for motor outcome accounting for gestational age included being small for gestational age, severe intraventricular hemorrhage, white matter injury volume, and chronic lung disease.

**Conclusions and Relevance:**

At preschool age, cognitive outcome was comparably associated with maternal education and neonatal brain injury. The association of brain injury with poorer cognition was attenuated in children born to mothers of higher education level, suggesting opportunities to promote optimal outcomes.

## Key Points

**Question:**

Does the association of brain injury with adverse neurodevelopmental outcome in preterm neonates vary by the socioeconomic status of the parents?

**Findings:**

In this cohort study of 226 preterm neonates, cognitive and motor outcomes were associated with different prenatal and postnatal clinical factors, with maternal education and brain injury having similar effect sizes for cognitive outcomes. Importantly, cognitive scores in preterm children in the higher-status group did not differ between those with and without brain injury.

**Meaning:**

Maternal education is associated with cognitive outcome in preterm neonates, with higher status appearing to attenuate the association of brain injury with neurodevelopmental outcome.

## Teaser

This cohort study evaluates the association of maternal education and brain injury with neurodevelopmental outcomes in children born very prematurely in Canada.

## Introduction

While improved intensive care therapies have increased the survival of critically ill neonates, preterm birth remains a leading cause of lifelong neurodevelopmental disability globally and in North America.^1,2,3,4,5,6^ While we understand much more about the association of brain injury with neurodevelopmental disability, we know far less about the role of environments and experiences in moderating these associations. In this article, we examine the association of neurodevelopment with brain injury in the context of socioeconomic status (SES), a factor that previous literature suggests systematically patterns environments and experiences of children.

Neurodevelopmental disabilities in preterm neonates are associated with white matter injury (WMI) and severe intraventricular hemorrhage (IVH).^6,7^ The role of experiential factors, such as SES, in mitigating or exacerbating risk for negative outcomes in preterm infants with brain injuries is incompletely understood. However, the neuroscience literature suggests many examples through which enriched environments can mitigate the impact of early-life brain injuries.^8,9,10,11,12^

In normative populations, many studies demonstrate associations between SES, as measured by maternal education, and poorer cognitive development, language skills, and academic achievement.^13,14,15,16,17,18,19,20^ In children born preterm, similar associations are recognized.^13,14,21^ Importantly, however, specific interventions to address these issues that are beneficial in children born at term are not necessarily effective in preterm-born children.^22^ This discrepancy may reflect the existence of brain injury in the preterm population. Furthermore, studies of children born preterm with brain injury have not sensitively accounted for the contribution of SES to neurodevelopmental outcomes or have considered SES without contemporary measures of brain injury.^23,24,25^

We sought to address the hypothesis that high SES, reflected by maternal education, would mitigate the association of brain injury with adverse neurodevelopmental outcome in the preterm neonate. We addressed this hypothesis in a prospective cohort of preterm neonates studied in Vancouver, Canada, a high-resource setting with a single-payer provincial health care system with uniform access to health care services across the range of SES.

## Methods

The University of British Columbia Clinical Research Ethics Board approved this study. Parental written informed consent was given following the recommendations of the board. This study follows the Strengthening the Reporting of Observational Studies in Epidemiology (STROBE) reporting guideline for cohort studies.^26^

### Study Population

Newborns were eligible if they were born between 24 and 32 weeks’ gestational age (GA). As reported previously,^27^ exclusion criteria were (1) congenital malformation or syndrome, (2) antenatal congenital infection, or (3) large periventricular hemorrhagic infarction (>2 cm) on clinical ultrasonography.

Study participants were recruited prospectively from August 6, 2006, to September 9, 2013, at the British Columbia Women's Hospital, the major provincial tertiary-level neonatal intensive care unit (NICU) (eFigure 1 in the Supplement). From January 2011 until the end of enrollment, with a change in clinical practice and grant funding, we only enrolled newborns exposed antenatally to magnesium sulfate for fetal neuroprotection or for the treatment of maternal preeclampsia. Some data from the same cohort at earlier ages of follow-up were described previously.^7,27,28,29,30,31,32,33^

### Clinical Data Collection

Systematic detailed medical record reviews were performed for clinical information about pregnancy, delivery, and NICU course. For this study, we focused on previously recognized factors associated with neurodevelopmental outcome in this population (Table 1).^1,7,29,34^ Chronic lung disease (CLD) was defined as the need for oxygen therapy at 36 weeks’ postmenstrual age.

**Table 1.** Clinical Factors and 4.5 Year Outcomes by Level of Maternal of Education

Clinical Factor	Maternal Level of Education, No./Total No. (%)			*P* Value
	Primary or Secondary School (n = 29)	Undergraduate Degree (n = 134)	Postgraduate Degree (n = 34)	
Prenatal				
Maternal age, median (IQR), y	30.5 (27-36.8)	32.1 (28.3-36.9)	32.4 (30.6-36.6)	.28
Preeclampsia	7/29 (24.1)	36/134 (26.9)	6/34 (17.7)	.51
Gestational diabetes	4/29 (13.8)	11/134 (8.2)	0	.40
Prenatal steroids	25/29 (86.2)	123/134 (91.8)	29/34 (85.3)	.30
Prenatal magnesium sulfate	8/29 (27.6)	27/134 (20.2)	6/34 (17.7)	.66
Histological chorioamnionitis	12/27 (44.4)	47/132 (35.6)	12/33 (36.4)	.69
Small for gestational age	7/29 (24.1)	24/134 (17.9)	3/34 (8.8)	.25
Birth and neonatal intensive care unit course				
Male	17/29 (58.6)	71/134 (53.0)	18/34 (52.9)	.78
Gestational age, mean (SD), wk	28.0 (2.1)	27.9 (2.2)	28.4 (2.6)	.53
Birth weight, mean (SD), g	1046.7 (303.6)	1038.3 (318.7)	1132.4 (347.9)	.31
Total ventilation, median (IQR), d	5.5 (1.5-37.0)	7.0 (1.0-35.0)	2.0 (1.0-29.0)	.60
Chronic lung disease, defined as oxygen at 36 weeks’ postmenstrual age	5 (16.7)	25 (18.7)	7 (20.6)	.69
Retinopathy of prematurity	12/24 (50.0)	51/109 (46.8)	7/27 (25.9)	.11
Postnatal infection	14/29 (48.3)	76/133 (57.1)	14/34 (41.2)	.23
Postnatal dexamethasone	7/29 (24.1)	24/134 (17.9)	8/33 (24.2)	.60
Punctate white matter injury present	11/29 (37.9)	37/134 (27.6)	8/34 (23.5)	.49
Punctate white matter injury volume, median (IQR), mm^3^	39.4 (20.3-95.5)	64.0 (19.5-431.9)	28.2 (15.2-253.9)	.56
Severe intraventricular hemorrhage (grade 3 or periventricular hemorrhagic infarction >2 cm)	1/26 (3.9)	3/128 (2.4)	2/33 (6.1)	.59
Outcome at 4.5 y				
Full-scale IQ, mean (SD)	99.6 (13.6)	101.0 (15.7)	108.3 (9.5)	.02
Wechsler Primary and Preschool Scale of Intelligence verbal IQ, mean (SD)	99.0 (15.5)	100.5 (16.1)	108.4 (12.2)	.02
Wechsler Primary and Preschool Scale of Intelligence performance IQ, mean (SD)	103.8 (11.2)	103.7 (13.8)	107.5 (8.1)	.01
Wechsler Primary and Preschool Scale of Intelligence processing speed, mean (SD)	94.4 (9.9)	96.8 (14.5)	100.7 (10.4)	.09
Movement Assessment Battery for Children percentile score, median (IQR)	25 (5-50)	25 (2-63)	56 (16-91)	.04

Abbreviation: IQR, interquartile range.

### Socioeconomic Status

We used maternal education at the time of NICU admission as our primary measure of SES^35,36^ given prior literature to support this being the most informative measure of SES in children born preterm in Canada.^37^ Taking the number of years of education into account, we categorized the level of maternal education as primary or secondary school, undergraduate degree, or postgraduate degree. We also collected paternal years of education and the occupation of both parents.

### Magnetic Resonance Imaging Studies

Detailed imaging methods applied in this cohort have been described previously.^29,30^ Using an isolette compatible with magnetic resonance imaging (MRI) (Lammers Medical Technology) and specialized neonatal head coil (Advanced Imaging Research), newborns underwent MRI first within several weeks after birth, as soon as they were deemed clinically stable, and again at term-equivalent age. We performed MRI studies without pharmacologic sedation on a Siemens 1.5-T Avanto scanner with 3-dimensional coronal volumetric T1-weighted images and axial fast spin echo T2-weighted images. An experienced neuroradiologist who was blinded to the newborn's medical history rated the severity of IVH, classifying severe IVH as grade 3 or periventricular hemorrhagic infarction larger than 2 cm.^38^ White matter injury volume was calculated as described previously.^7^

### Neurodevelopmental Outcomes

#### Ages 18 Months and 36 Months 

At 18 months’ corrected age, children underwent a first neurodevelopmental assessment conducted by examiners unaware of MRI findings. Median (interquartile range [IQR]) age at this first examination was 18.65 (18.3-19.4) months. At 36 months’ corrected age (median [IQR] age, 35 [33.9-37.1] months), children underwent a second assessment. Examiners assessed neurodevelopment using the Bayley Scales of Infant and Toddler Development, Third Edition (Bayley-III),^39^ which yields composite scores (standardized with mean [SD] of 100 [15]) for cognitive and motor skills.

#### Age 4.5 Years 

At age 4.5 years (median [IQR] age, 4.8 [4.8-4.9] years), neurodevelopmental outcomes were assessed by PhD-trained psychology staff, plus 1 highly experienced master’s degree–level psychologist, at British Columbia Children's and Women's Hospitals.

The Wechsler Primary and Preschool Scale of Intelligence, Fourth Edition (WPPSI-IV)^40^ was performed to provide an overall full-scale intelligence quotient (FSIQ) as well as scores for verbal comprehension, perceptual reasoning, working memory, and processing speed. Overall indices are standard scores with mean (SD) of 100 (15).

Motor function was assessed using the Movement Assessment Battery for Children–Second Edition (M-ABC2),^41^ which assesses manual dexterity, aiming and catching, and balance domains. The total test score was standardized and converted to a percentile rank.^42^

### Statistical Analysis

Statistical analysis was performed using Stata statistical software version 15.0 (StataCorp LLC) during 2018 when the 4.5-year outcome measures were available for the whole cohort. Clinical characteristics were compared by maternal level of education using 2-tailed Fisher exact tests and Kruskal-Wallis tests for categorical and continuous data, respectively. A nonparametric test for trends across ordered groups was performed to examine the association of maternal and paternal level of education.

Using multivariable linear models to account for GA, we examined clinical variables associated with M-ABC2 and FSIQ at 4.5 years. Prenatal and postnatal clinical variables examined are listed in Table 1. Selection of the best model was performed through the method of all possible equations, which identifies the best subset for linear regression.^43^ We then assessed our final models using generalized estimating equations using an independent correlation structure with robust standard errors estimation to account for correlation within twin pairs in the cohort. We then performed mixed linear regression models to test the selected model for the longitudinal measurements of cognition (18- and 36-month Bayley-III and 4.5-year WPPSI-IV FSIQ) and mixed-effects logistic regression analysis for the longitudinal trajectory of motor function (eMethods in the Supplement). A *P* value less than .05 was considered statistically significant. A detailed description of the missing data approach, including multiple imputation, can be found in the eMethods in the Supplement.

## Results

Of 226 survivors, 88% (199 children; 96 [48.2%] female) were assessed at 18 months, 83% (187 children; 90 [48.1%] female) at 36 months, and 75% (170 children; 80 [47.1%] female) at 4.5 years. Two hundred eight children (92%) in the cohort had at least 1 assessment, 191 (85%) had 2, and 157 (70%) had all 3. For a detailed cohort flow, see eFigure 1 in the Supplement. Available and missing values on cognitive outcomes are described in eTable 1 in the Supplement.

### SES and Clinical Factors

Maternal level of education was available for 197 patients (84.2%). Approximately two-thirds of the mothers completed an undergraduate degree (Table 1). Prenatal and postnatal characteristics did not differ between the groups of maternal levels of education. Maternal level of education was associated with paternal level of education (*z* = 5.33; *P* = .001). In only 3 children, the maternal level of education changed from NICU admission to the 4.5-year assessment: 2 in the undergraduate degree group obtained postgraduate degrees and 1 in the secondary education group obtained an undergraduate degree. Agreement of the cognitive scores at different points is shown in eTable 2 in the Supplement.

### Maternal Level of Education and Outcome Measures

#### Cognitive Outcome

**Figure 1.**
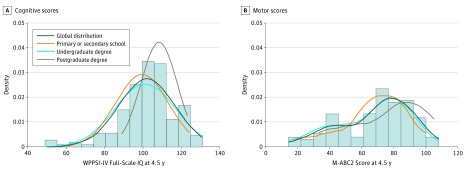
Distribution of Cognitive and Motor Scores at 4.5 Years by Maternal Level of Education Distribution of cognitive (A) and motor (B) scores at age 4.5 years across the cohort divided by maternal level of education. The postgraduate group achieved a mean IQ and a median Movement Assessment Battery for Children (M-ABC2) score higher than the undergraduate degree group and primary or secondary school group (*P* = .01 and *P* = .02, respectively). Bars indicate the distribution of the studied variable for the whole cohort. Lines indicate the distribution of the studied variable by maternal level of education. WPPSI-IV indicates Wechsler Primary and Preschool Scale of Intelligence, Fourth Edition.

The mean (SD) FSIQ at 4.5 years was 102.0 (14.6). The mean (SD) FSIQ score in children whose mothers had a postgraduate degree (108.30 [9.45]) was significantly higher than the mean (SD) FSIQ scores of both the undergraduate degree group (98.66 [19.65]; *P* = .01) and the primary or secondary school group (96.24 [19.96]; *P* = .02); the mean FSIQ scores of the undergraduate and primary or secondary school groups did not differ (Figure 1).

#### Motor Outcome

The median (IQR) M-ABC2 percentile score at age 4.5 years was 37 (5-63). The median (IQR) M-ABC2 percentile score was significantly higher in children whose mothers had a postgraduate degree (56.5 [16-91]) than in those in the undergraduate degree group (25 [2-63]) (*P* = .03) (Figure 1).

### Clinical Factors Associated With the 4.5-Year Outcome and Longitudinal Neurodevelopmental Outcomes

#### Cognitive Outcome

The best model for cognitive outcome tested using the WPPSI-IV FSIQ at 4.5 years, adjusting for GA, included severe IVH, WMI volume, CLD, and maternal level of education. When examining maternal level of education, with undergraduate degree as the reference category, the cognitive outcome of the postgraduate degree group was higher (β = 7.13; 95% CI, 1.53-12.73; *P* = .01) (Table 2). (See eTable 3 and eTable 4 in the Supplement for a detailed description of missing values and the estimated model obtained after performing multiple imputation.) The cohort included 38 twin pairs. Using generalized estimating equations to account for twin pairs in the data structure resulted in a strengthening of these coefficients.

**Table 2.** Estimated Models of Wechsler Primary and Preschool Scale of Intelligence Full-Scale IQ Score and Movement Assessment Battery for Children Percentile Score at 4.5 Years

Factor	β Coefficient (95% CI)	*P* Value	Standardized β	Accounting for Twin Pairs (n = 38 Pairs)	
				β Coefficient (95% CI)	*P* Value
Cognitive (n = 162)^a^					
Gestational age	0.50 (−0.53 to 1.53)	.34	0.05	0.30 (−0.97 to 1.57)	.64
Chronic lung disease	−7.89 (−13.81 to −1.97)	.009	0.17	−7.96 (−15.24 to −0.67)	.03
Severe intraventricular hemorrhage(grade 3)	−9.69 (−21.29 to 1.90)	.10	0.23	−23.5 (−37.76 to −9.23)	.001
Punctate white matter injury volume	−0.01 (−0.01 to −0.003)	.001	0.23	−0.01 (−0.02 to −0.004)	.001
Maternal level of education					
Primary or secondary school	−1.48 (−7.78 to 4.83)	.64	0.21	−2.63 (−10.39 to 5.13)	.51
Undergraduate degree	1 [Reference]	1 [Reference]		1 [Reference]	1 [Reference]
Postgraduate degree	7.13 (1.53 to 12.73)	.01		10.14 (3.26 to 17.03)	.004
Motor (n = 159)^b^					
Gestational age	3.01 (0.67 to 5.35)	.01	0.21	3.01 (0.74 to 5.28)	.009
Chronic lung disease	−18.70 (−32.05 to 5.35)	.006	0.22	−18.70 (−31.70 to −5.71)	.005
Severe intraventricular hemorrhage(grade 3)	−37.43 (−65.24 to −9.62)	.009	0.20	−37.43 (−64.50 to −10.37)	.007
Punctate white matter injury volume	−0.03 (−0.05 to −0.01)	.003	0.21	−0.03 (−0.04 to −0.01)	.002
Small for gestational age	−17.62 (−29.9 to −5.35)	.005	0.21	−17.62 (−29.56 to −5.67)	.004

^a^ Cognitive model: *F*_5,156_ = 6.07; *P* < .001. Mallow Cp statistic: 4.84; adjusted *R*^2^=0.14; Akaike information criteria: 1355.5; Schwarz Bayesian criterion: 1373.0. See eMethods in the Supplement for a detailed description of this model after multiple imputation (n = 170).

^b^ Motor model: *F*_5,137_ = 9.43; *P* < .001; Mallow Cp statistic: 0.72; adjusted *R*^2^=0.21; Akaike information criteria: 1189.9; Schwarz Bayesian criterion: 1206.0.

When examining the standardized β coefficients, maternal level of education (standardized β = 0.21) had an association with cognitive outcomes that was similar to that of WMI (standardized β = 0.23) and severe IVH (standardized β = 0.23), and greater than that of CLD (Table 2).

When SES was operationalized by other factors (eg, years of education, occupation), associations of our primary clinical conditions with cognitive outcome at 4.5 years were similar (eTable 5 in the Supplement). The same model was tested for the 18-month and 36-month cognitive outcomes (eTable 6 in the Supplement).

#### Cognitive Trajectory 18 Months, 36 Months, and 4.5 Years

Cognitive scores were positively associated with increasing maternal level of education and negatively associated with brain injury and CLD at all points using mixed-effects models to examine the association with cognitive scores longitudinally (eTable 7 in the Supplement).

#### SES Modifies the Association Between Brain Injury and Outcome in Children Born Preterm

**Figure 2.**
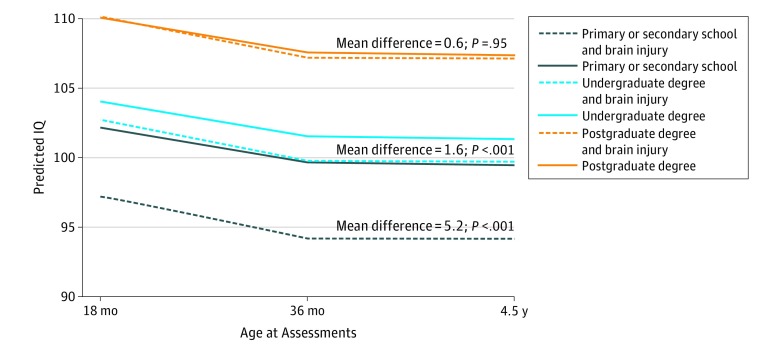
Estimated IQ in Neonates With and Without Brain Injury by Maternal Level of Education Mean difference of IQ scores by groups of maternal level of education.

When comparing cognitive scores in children with and without brain injury (Table 3 and Figure 2), SES was associated with modified cognitive outcomes of both groups of children. In the absence of brain injury, the higher SES group achieved a predicted FSIQ that is 7.4 points higher (95% CI, 6.99-8.83; *P* < .001) than the lower SES group. In the presence of brain injury, the association with SES increased, with the higher SES group having a mean increase of 13.7 points (95% CI, 13.34-14.25; *P* < .001) relative to the lower SES group.

**Table 3.** Estimated Cognitive Scores in Children With and Without Brain Injury at Different Points

Variable	No Brain Injury^a^		Brain Injury^b^	
	Cognitive Score, Mean (95% CI)	*P* Value	Cognitive Score, Mean (95% CI)	*P* Value
Cognitive score at 18 mo^c^	102.2 (96.3 to 108.2)	.001	97.2 (89.1 to 105.3)	.001
Cognitive score at 36 mo^c^	99.7 (93.7 to 105.7)	.001	94.2 (86.1 to 102.4)	.001
Cognitive score at 4.5 y^c^	99.5 (93.5 to 105.5)	.001	94.2 (85.9 to 102.5)	.001
Gestational age (centered at 28 wk)	1.3 (0.5 to 2.1)	.002	0.3 (−1.3 to 1.9)	.69
Maternal education				
Primary or secondary school^d^	[Reference]	[Reference]	[Reference]	[Reference]
Undergraduate degree^d^	2.0 (−4.3 to 8.3)	.54	5.6 (−3.6 to 14.8)	.23
Postgraduate degree^d^	7.4 (0.01 to 14.8)	.05	13.7 (2.2 to 25.1)	.02

^a^ The group with no brain injury included 344 observations and 124 patients. Log likelihood = −1321.17; *P* = .001.

^b^ The group with brain injury included 170 observations and 62 patients. Log likelihood = −648.38; *P* = .001.

^c^ Mean (95% CI) of the cognitive scores at each occasion.

^d^ Associated increment in cognitive scores with maternal level of education.

When comparing the estimated cognitive scores of children in different SES groups, brain injury exhibited a nonuniform association. In the higher SES group, the presence of brain injury was not associated with FSIQ (absolute mean difference = 0.7; 95% CI, −1.4 to 0.1; *P* = .09); the association of brain injury and FSIQ was stronger across the undergraduate group (absolute mean difference = 1.6; 95% CI, 1.2-1.9; *P* < .001) and the primary or secondary school group (absolute mean difference = 5.2; 95% CI, 4.6-5.9; *P* < .001) (Figure 2 and eFigure 2 in the Supplement).

### Motor Outcome at 4.5 Years

The best model for motor outcome (measured using the M-ABC2 percentile score) at 4.5 years, adjusting for GA, included being small for GA and having severe IVH, WMI volume, and CLD (*F*_5,137_ = 9.43; *P* < .001; *R*^2^_Adj_ = 0.21). When examining the standardized β coefficients, these 3 variables exhibited similar associations to motor outcomes (Table 2). Maternal level of education was not associated with motor outcome when included in this final model (β = 4.02; 95% CI, −1.23 to 9.27; *P* = .24). This model applied to the longitudinal motor scores is described in eTable 8 in the Supplement.

## Discussion

In this study of children born very preterm, maternal education, brain injury, and CLD were the most significant factors associated with cognitive outcome at preschool age. Maternal education, an important indicator of SES, had the same statistical association with cognitive outcome as brain injury, including quantitative measures of WMI. Importantly, the association of brain injury and CLD with cognitive outcome was modified by SES such that these postnatal complications were not found to be associated with lower FSIQ in preterm children born to mothers with postgraduate education. Prior literature indicates an association of social inequities with brain maturation and neurodevelopmental outcomes in children; however, previous studies typically addressed SES in the context of poverty.^18,19,44,45^ The present study evaluated the role of SES in a relatively affluent geographical location with universal medical care. Our findings highlight the potential of higher SES to mitigate the consequence of neonatal brain injury in children born preterm.

Maternal education did not have the same association with motor outcomes, a finding consistent with previous studies.^46^ Motor outcomes were significantly associated with lower GA at birth, GA at birth, developing CLD, and brain injury. At age 4.5 years, the best models for motor and cognitive outcomes suggest some distinction in the pathways mediating these important aspects of neurodevelopmental outcome. These findings also reinforce the importance of prenatal and postnatal risk factors when considering interventions to improve neurodevelopment of children born preterm.^47,48^

Although the nature of preterm brain injury has evolved over the past decades, its association with neurodevelopmental outcome remains strong. Despite advances in perinatal care, cognitive outcomes among children born preterm have not improved since the 1990s.^2,3,49,50^ Preterm-born children still perform almost 1 SD below full-term children on intelligence tests.^2^ With the decline of cystic periventricular leukomalacia,^51^ the implications of the more recently recognized patterns of injury, such as punctate WMI, are shifting our focus to modifiable risk factors that might allow clinicians and parents to enhance brain development and improve functional outcomes.

Even in normative populations, contemporary brain imaging studies have demonstrated the association of SES factors with brain structure and development.^44,52,53^ For example, in a study^44^ of 1500 children in Los Angeles County, parental education was robustly associated with children’s total brain surface area. It has been suggested that the developing brain’s capacity for repair provides opportunities for interventions to dampen or even reverse the effects of early adversity, whether environmental or medical. The beneficial effects of early childhood intervention programs on later function are promising.^54,55^ In the Carolina Abecedarian Project, children from low-income families who were randomized to receive early educational intervention had greater developmental and education achievements^56^ and a lower prevalence of cardiovascular and metabolic disease in their mid-30s.^54^ Importantly, attempts to replicate these findings in children born preterm have not been as successful.^22^ Other interventions based on social risk have yielded better early cognition in preterm infants.^57^ Observational studies in preterm infants suggest a beneficial association between early cognition and interventions that promote sensitive parenting^58^ and increase access to resources such as high-quality day care.^59^ Together with the strong effect sizes of the Carolina Abecedarian trial and our new data, these findings support the importance of early experience and education to promote optimal neurodevelopmental outcomes following early-life adversity.

A possible explanation for our findings is that mothers with postgraduate education confer genes for higher intelligence to their offspring. In a recent meta-analysis, only up to 4.8% of the variance in intelligence is explained by genome-wide association study results.^60^ Furthermore, the genetic contributions to cognition appear to differ across SES in some cohorts. For example, in one study, the heritability of IQ is estimated at 5% in low-SES families, but up to 50% in high-SES families.^61^ In contrast, in a large representative cohort of twins in the United Kingdom, the estimated association of genetic factors with intelligence was similar in low- and high-SES families.^62^ In a sample of 11 000 pairs of twins from 4 countries, heritability of general cognitive ability was correlated with age: 41% at age 9 years to 66% at age 17 years.^63^ In children born at extremely low GA, those whose mothers received advanced education demonstrated improved inhibitory control and processing speed at age 10 years.^13^ Taken together, these findings suggest that the outcomes observed in our cohort of preschool children are not explained by genetic factors alone.

Robust neuroscience studies in animal models demonstrate that animals raised in enriched environments that include social stimulation, exercise, and novelty have improved brain structure and functional outcomes, including learning, memory, and plasticity.^64,65,66,67,68^ In a rodent model of neonatal seizures, even following status epilepticus, an enriched environment enhanced cognitive function, likely through an increase in neurogenesis.^12^ These experimental findings are congruent with our finding of higher SES modifying the association of early-life brain injury with preschool age cognitive outcome. In our clinical study, we used maternal education at the time of NICU admission as our primary measure of SES. When examining associations with other measures of SES, consistent with a prior study across Canadian centers, we found that maternal education is the most robust measure.^37^ The urgent challenge is now to identify the specific elements that differ in the experience of children born to mothers with higher educational attainment so that effective and efficient interventions can be evaluated.

Our findings also highlight the importance of postnatal illness as a contributor to neurodevelopmental outcomes in children born preterm. Postnatal comorbidities, including infections, retinopathy of prematurity, and CLD, as well as brain injury,^34,50,69^ are recognized to have negative associations with the long-term outcome of this population. In our study, together with brain injury, CLD was the most important postnatal comorbidity associated with cognitive outcomes. This finding is consistent with the association of CLD with white matter maturation at term equivalent age^70^ and long-term cognitive outcomes of preterm children.^2^ The importance of CLD is particularly reinforced in this cohort, in which the incidence of CLD (18%) was lower than the 27% incidence reported in the literature.^2,50,71,72,73,74,75,76^ Importantly, SES was associated with cognitive outcomes even when accounting for brain injury and CLD. Our findings are also consistent with earlier observations in this cohort that optimal parent-infant interaction might mitigate neurodevelopmental consequences of early-life procedural pain.^77^ Together, these findings highlight the potential to favorably affect trajectories of neurodevelopmental outcome by reducing exposure to early-life morbidities (eg, CLD) and enhancing the postnatal experience.

While prior studies of preterm children demonstrated that intelligence is negatively associated with GA,^3,49,78^ in our cohort, GA is only an important factor associated with outcome in those without brain injury. In the presence of brain injury and CLD, GA was not independently associated with cognitive outcomes. This finding is consistent with a recent meta-analysis^2^ in which the association between CLD and outcome was not confounded by GA, and with a brain imaging study^79^ of preterm neonates in which the association of very preterm birth with adverse white matter maturation was mediated by postnatal illness rather than extreme preterm birth itself. These findings reinforce that GA is not a fixed determinant of neurodevelopmental outcome and instead acts through potentially modifiable comorbidities that explain the wide variability in neurodevelopmental outcomes among children born at the same GA.

### Limitations

Our study has several limitations. Data on maternal level of education were missing in 14% of the cohort, creating the potential for selection bias.^14^ Nevertheless, it is likely that this potential selection bias would have resulted in underestimation of the association of SES with cognitive outcome because children lost to follow-up tend to be from lower SES groups.^80^ If those in the lower maternal education group who were seen in follow-up were the most organized, we would expect the differences observed in our study to be further attenuated. We also recognize the use of different cognitive scores at ages 1.5 and 3 years relative to 4.5 years. Prior evidence^81^ and the intraclass correlation coefficients support the use of these scores in a longitudinal analyses. Assessment of cognition in young children becomes more complete with age, as more domains can be assessed.^24,81^ Thus, some change in scores within an individual may reflect better assessment of cognition rather than differences in the scoring tool. Our cohort was recruited from a high-resource setting with uniform access to health care. Further research could help identify determinants in more heterogeneous settings. In British Columbia, although there is universal coverage for health care and early intervention services, access to allied health care services may vary by region. Given available data, we were unable to adjust for individual patient interventions. We found a tight correlation of maternal and paternal education and occupation. As such, we did not distinguish specific factors associated with maternal rather than paternal education contributing to cognitive outcomes of children born preterm.

## Conclusions

In children born preterm, preschool age cognitive outcome was associated with maternal level of education, an important measure of SES, with an effect size similar to that of neonatal brain injury. The association of brain injury with adverse cognitive outcomes in children born preterm was attenuated in children born to mothers of higher education level. While we recognize that both brain injury and lower SES were associated with neurodevelopmental disability in children born preterm, we found that both of these issues are of comparable importance and have the potential to influence each other. Our findings highlight that the neurodevelopmental sequelae of preterm birth are not static, but rather evolve through early childhood, offering the potential for modification by factors such as brain injury and SES. Future studies are needed to identify which aspects of higher SES have the greatest association with cognitive outcomes so that potential interventions and policies can be optimized for beneficial impact.

## Notes

Supplement. **eMethods.** Statistical Analysis **eFigure 1.** Cohort Flow **eFigure 2.** Predicted IQ According to Maternal Level of Education and the Presence of Brain Injury (Adjusted for GA) **eTable 1.** Missing Values on the Dependent Variables **eTable 2.** Agreement of the Cognitive Scores at Different Time Points **eTable 3.** Missing Data of Independent Variables in the Model Related to Cognitive Scores at 4.5 Years (n=170) as Dependent Variable **eTable 4.** Estimated Model After Multiple Imputation **eTable 5.** Other Indexes of SES and Maternal Ethnicity Tested as Independent Variables With White Matter Injury, Severe Intraventricular Hemorrhage, Chronic Lung Disease and Gestational Age at Birth for the Prediction of FSIQ at 4.5 Years **eTable 6.** Regression Model Obtained for the 4.5 Year-Outcome Final Model Applied to the 18 and 36 Months Cognitive Outcome **eTable 7.** Estimated Cognitive Outcome Across Different Assessment Times Through Mixed Effects Models **eTable 8.** Estimated Odds Ratio (OR) and 95% Confidence Intervals of the Motor Outcome Associated Variables Across Time Through Mixed-Effects Logistic Regression **eReferences**
