# Supplementary material for: Association of Socioeconomic Status and Brain Injury With Neurodevelopmental Outcomes of Very Preterm Children
Source: JAMA Netw Open. 2019 May 3;2(5):e192914. doi: 10.1001/jamanetworkopen.2019.2914 (PMC6503490; doi:10.1001/jamanetworkopen.2019.2914)
Supplement: Supplement. — eMethods. Statistical Analysis eFigure 1. Cohort Flow eFigure 2. Predicted IQ According to Maternal Level of Education and the Presence of Brain Injury (Adjusted for GA) eTable 1. Missing Values on the Dependent Variables eTable 2. Agreement of the Cognitive Scores at Different Time Points eTable 3. Missing Data of Independent Variables in the Model Related to Cognitive Scores at 4.5 Years (n=170) as Dependent Variable eTable 4. Estimated Model After Multiple Imputation eTable 5. Other Indexes of SES and Maternal Ethnicity Tested as Independent Variables With White Matter Injury, Severe Intraventricular Hemorrhage, Chronic Lung Disease and Gestational Age at Birth for the Prediction of FSIQ at 4.5 Years eTable 6. Regression Model Obtained for the 4.5 Year-Outcome Final Model Applied to the 18 and 36 Months Cognitive Outcome eTable 7. Estimated Cognitive Outcome Across Different Assessment Times Through Mixed Effects Models eTable 8. Estimated Odds Ratio (OR) and 95% Confidence Intervals of the Motor Outcome Associated Variables Across Time Through Mixed-Effects Logistic Regression eReferences [file jamanetwopen-2-e192914-s001.pdf]

Supplementary Online Content

Benavente-Fernández I, Synnes A, Grunau RE, et al. Association of socioeconomic status and brain injury with neurodevelopmental outcomes of very preterm children. *JAMA Netw Open*. 2019;2(5):e192914. doi:10.1001/jamanetworkopen.2019.2914

eMethods. Statistical Analysis
eFigure 1. Cohort Flow
eFigure 2. Predicted IQ According to Maternal Level of Education and the Presence of Brain Injury (Adjusted for GA)
eTable 1. Missing Values on the Dependent Variables
eTable 2. Agreement of the Cognitive Scores at Different Time Points
eTable 3. Missing Data of Independent Variables in the Model Related to Cognitive Scores at 4.5 Years (n=170) as Dependent Variable
eTable 4. Estimated Model After Multiple Imputation
eTable 5. Other Indexes of SES and Maternal Ethnicity Tested as Independent Variables With White Matter Injury, Severe Intraventricular Hemorrhage, Chronic Lung Disease and Gestational Age at Birth for the Prediction of FSIQ at 4.5 Years
eTable 6. Regression Model Obtained for the 4.5 Year-Outcome Final Model Applied to the 18 and 36 Months Cognitive Outcome
eTable 7. Estimated Cognitive Outcome Across Different Assessment Times Through Mixed Effects Models
eTable 8. Estimated Odds Ratio (OR) and 95% Confidence Intervals of the Motor Outcome Associated Variables Across Time Through Mixed-Effects Logistic Regression
eReferences

This supplementary material has been provided by the authors to give readers additional information about their work.

© 2019 Benavente-Fernández I et al. *JAMA Network Open.*

eMethods

Statistical analysis.

Selection of the best predictive model for 4.5-year WPPSI-IV FSIQ was performed through the method of "all possible equations," which aims to find the best subset for linear regression. We allowed non-hierarchical submodels. All possible combinations of the independent variables were computed and evaluated to find a set of parameters for each subset. Mallow's Cp, Akaike Information Criteria (AIC), Schwarz Bayesian Criterion (BIC), R-squared (R2) and adjusted R-squared (R2Adj) were computed for each subset. With this method, we tested all the prenatal and postnatal clinical factors (SGA, GA, birth weight, magnesium sulfate administration, prenatal steroids, preeclampsia, gestational diabetes, histological chorioamnionitis, sex, severe IVH, punctate WMI volume, CLD, total ventilation days, retinopathy of prematurity, postnatal infection and postnatal cumulative dose of dexamethasone) and maternal level of education. Internal validation of the model was performed with cross-validation on the specified models in order to evaluate the model's ability to fit out-of-sample data. Standardized beta coefficients were estimated to compare the effect size of the different predictors of outcome. These are the regression coefficients obtained by first standardizing all variables to have a mean of 0 and a standard deviation of 1.

We then performed mixed linear regression models to test the selected model to the longitudinal measurements of cognitive IQ estimation (18- and 36-month Bayley-III and 4.5-year WPPSI-IV FSIQ) accounting for repeated measurements. We tested whether different ages at assessment would significantly change the slope of the cognitive scores. For that purpose, we compared the random slope and random intercept models using a likelihood ratio test of the null hypothesis $H0$: $\sigma u12=\sigma u01=0$. We accepted the null and select the random intercept model over the random slope model. Different ages at assessment did not meaningfully change the slope of cognitive scores. Allowing slopes to vary across individuals does not bring the predicted values closer to the observed values of cognitive scores for each individual. We also tested the interaction of maternal level of education with age at assessment, which was not significant. We therefore treated time as a categorical variable in the multivariate model. The obtained model treats an individual's repeated measurements of cognitive scores as a multivariate response giving us the parameters for the mean and variance of these scores at each occasion.

We performed the same stepwise approach to the motor outcome and once we found the best subset predictive of 4.5-year motor outcome (M-ABC2 centile rank), we then studied the longitudinal trajectory through. Since Bayley-III motor scores at 18 months and 36 months scale and standardization measurements differ from M-ABC2 percentile scores, when studying the longitudinal motor trajectory, we applied the best predictive model obtained at 4.5 years to the dichotomized motor outcome (motor outcome was considered adverse if M-ABC2 percentile score at 4.5 years ≤15 or if Bayley-III motor score ≤ 85 at 18 and 36 months).

Supplemental figures:

© 2019 Benavente-Fernández I et al. *JAMA Network Open.*

eFigure1. Cohort flow

Parents of eligible children occurred following pre-term birth and NICU admission. Approach was based on inclusion and exclusion criteria; the diagnosis of brain injury was based on the MRI scans that followed enrolment.

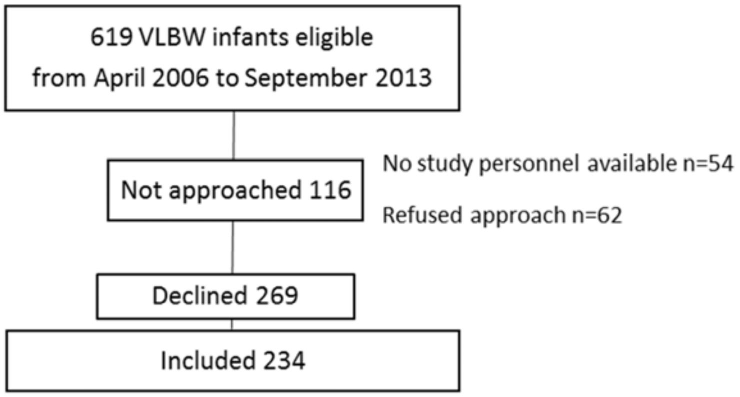

	Included	Eligible but not included	
Gestational age	27.6 (2.2)	28.6 (2.2)	0.0001
Birth weight	1047.9 (323.0)	1211.1 (381.7)	0.0001
Sex (male)	120 (51.3)	64 (55.2)	0.493

eFigure 2. Predicted IQ according to maternal level of education and the presence of brain injury (adjusted for GA). The difference in neonates with and without brain injury is attenuated in children born to mothers with a postgraduate degree level of education

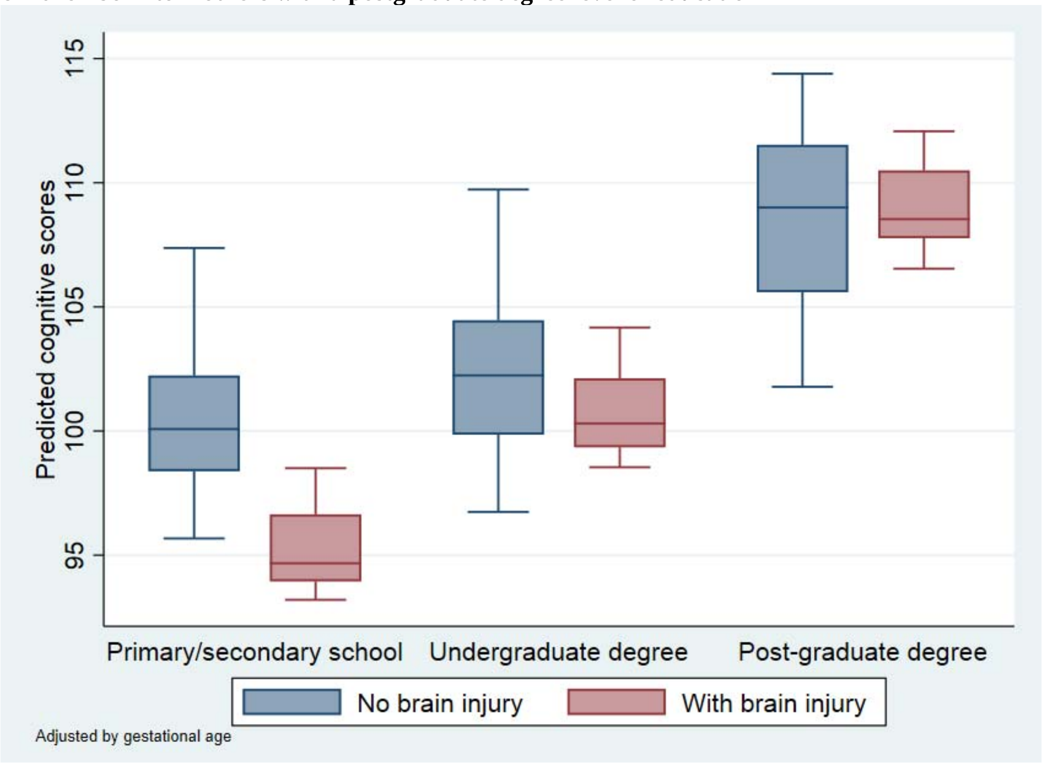

© 2019 Benavente-Fernández I et al. *JAMA Network Open.*

Supplemental tables.

Missing values description and approach

eTable 1. Missing values on the dependent variables

Cognitive outcomes	Valid data (%)	Missing data (%)
Cognitive score at 18 months	199 (85.04)	35 (14.96)
Cognitive score at 36 months	187 (79.91)	47 (20.09)
Cognitive score at 4.5 years	170 (72.65)	64 (27.35)

4.5-year cognitive outcome: potential selection bias given missing values[1].

Maternal level of education is known for 32 (50%) of the 64 patients with missing values on cognitive scores at 4.5 years with no distribution shift towards the lower SES group: 5 (15.6%) in primary/secondary school group, 23 (71.9%) in the undergraduate group and 4 (12.5%) in the post-graduate degree group.

eTable 2. Agreement of the cognitive scores at different time points

Agreement of cognitive scores	ICC_A	ICC_A CI 95 %	ICC_c	ICC_C CI 95 %
18 m (Bayley III)- 36 months (Bayley III)	0.77	0.69- 0.83	0.78	0.70- 0.83
33 months (Bayley III)- 4.5 years (WPPSI FSIQ)	0.69	0.58- 0.78	0.69	0.57- 0.78

Agreement of the cognitive scores at different time points (18 and 36 months, and 4.5 years) measured by intraclass coefficient (ICC_C) and absolute agreement intraclass coefficient (ICC_A)

© 2019 Benavente-Fernández I et al. *JAMA Network Open.*

Missing values on the independent variables

eTable 3. Missing data of independent variables in the model related to cognitive scores at 4.5 years (n=170) as dependent variable:

	Missing values
Maternal level of education	4/170 (2.35%)
Severe IVH	4/170 (2.35%)
Chronic lung disease	0
Punctate WMI volume	0
Gestational age	0

As illustrated in table 2 of the main manuscript, the model is based in 162 instead on 170 patients given 4 patients had no data on maternal level of education and 4 patients had no data on severe IVH. This table with a low rate of missing values is complemented by models applying multiple imputation for the independent variables (eTable 7).

eTable 4. Estimated model after multiple imputation

	β coeff (95% CI)	P
Gestational age	0.36 (-0.90- 1.62)	0.575
Chronic lung disease	-7.48 (-14.69 -0.26)	0.042
Severe IVH	-22.73 (-37.12 -8.35)	0.02
Punctate WMI volume	-0.01 (-0.02 -0.004)	0.001
Maternal level of education	6.37 (1.82- 10.92)	0.006

Number of obs= 170; $F(5, 162.0) = 6.42$; P= 0.0001

© 2019 Benavente-Fernández I et al. *JAMA Network Open.*

eTable 5. Other indexes of SES and maternal ethnicity tested as independent variables with white matter injury, severe intraventricular hemorrhage, chronic lung disease and gestational age at birth for the prediction of FSIQ at 4.5 years: Maternal SES has a stronger impact in cognitive outcome that paternal SES, regardless of the variable used to measure SES.

Index of SES	Coefficient	P value	Beta standardized coefficient
Maternal Hollingshead score	0.48	0.0001	0.31
Paternal Hollingshead score	0.24	0.025	0.17
Combined Hollingshead score	0.36	0.002	0.23
Maternal years of education	1.71	0.001	0.25
Paternal years of education	1.03	0.04	0.15
Maternal years of education and paternal years of education*	1.44	0.012	0.21
	0.41	0.465	0.06
Paternal level of education	3.02	0.039	0.16
Maternal ethnicity	0.02	0.982	0.002

We estimated the Hollingshead's four-factor score, derived from both education and occupation information[2]. Both parents were scored separately, and a combined score was also calculated.
Each variable was tested in a separate multivariate model for the prediction of FSIQ at 4.5 years of age, including the variables shown to better predict the outcome with SES: white matter injury, severe intraventricular hemorrhage and chronic lung disease and gestational age at birth. Here we only express the parameters that each SES index showed in the multivariate model.
*When including both maternal and paternal years of education as independent variables, only maternal years of education remained significant, the interaction term being non-significant (P=0.595).

© 2019 Benavente-Fernández I et al. *JAMA Network Open.*

eTable 6. Regression model obtained for the 4.5 year-outcome final model applied to the 18 and 36 months cognitive outcome: 18 month and 36 month cognitive outcomes evaluated given lower rates of missing values than the 4.5 year assessment

**Cognitive scores at 18 months**			
	β coeff (95% CI)	P	β st
Gestational age	0.57 (-0.36-1.50)	0.226	0.10
Chronic lung disease	-7.56 (-12.97- -2.15)	0.006	0.21
Severe IVH	-7.14 (-18.95- 4.68)	0.235	0.08
Punctate WMI volume	-0.01 (-0.01- -0.003)	0.001	0.25
Maternal level of education			
Primary/secondary school	-2.96 (-8.63- 2.71)	0.305	0.22
Undergraduate degree	Ref.	Ref.	
Postgraduate degree	7.59 (2.51-12.68)	0.004	
**Cognitive scores at 36 months**			
	β coeff (95% CI)	P	β st
Gestational age	0.40 (-0.58-1.38)	0.426	0.06
Chronic lung disease	-3.28 (-9.01- 2.46)	0.261	0.09
Severe IVH	-8.04 (-19.49- 3.41)	0.168	0.11
Punctate WMI volume	-0.009 (-0.01- -0.004)	0.0001	0.26
Maternal level of education			
Primary/secondary school	-7.26 (-13.47- -1.05)	0.022	0.24
Undergraduate degree	Ref.	Ref.	
Postgraduate degree	5.33 (-0.11-10.77)	0.055	

© 2019 Benavente-Fernández I et al. *JAMA Network Open.*

eTable 7 Estimated cognitive outcome across different assessment times through mixed effects models.

		Adjusting for GA only<br><br>Number of obs= 556;<br>Number of patients = 208;<br>Log likelihood= -2141.94.<br>P= <0.001		Adjusting for GA and maternal level of education<br><br>Number of obs= 537<br>Number of patients= 197<br>Log likelihood= -2068.1704<br>P= 0.0001		Adjusting for GA, maternal level of education, punctate WMI, severe IVH and CLD<br><br>Number of obs= 537<br>Number of patients= 197<br>Log likelihood= -2066.1076<br>P= 0.0001	
		Mean (95% CI) cognitive scores	P value	Mean (95% CI) cognitive scores	P value	Mean (95% CI) cognitive scores	P value
Cognitive score at 18 m		105.0 (103.1- 106.8)	0.001	104.5 (102.2- 106.7)	0.001	106.0 (103.6-108.4)	0.001
Cognitive score at 36 m		102.2 (100.3- 104.2)	0.001	101.9 (99.6- 104.2)	0.001	103.3 (100.9-105.8)	0.001
Cognitive score at 4.5 y		101.7 (99.7- 103.8)	0.001	101.5 (99.2- 103.9)	0.001	103.2 (100.7-105.7)	0.001
Gestational age (centered at a gestational age at birth of 28 weeks)		1.0 (0.3-1.8)	0.007	1.0 (0.3- 1.7)	0.01	0.6 (-0.2- 1.4)	0.17
Maternal level of education	Primary/secondary school			-4.1 (-9.0- 0.8)	0.10	-4.5 (-9.3- -0.4)	0.007
	Undergraduate degree			Ref.	Ref.	Ref.	Ref.
	Postgraduate degree			5.2 (0.6- 9.7)	0.03	6.4 (2.0-10.7)	0.004
Brain injury	Punctate WMI volume					-0.01 (-0.01- -0.004)	0.001
	Severe IVH					-7.7 (-17.1- 1.8)	0.11
Chronic lung disease						-5.9 (-10.5- -1.3)	0.01

GA: gestational age. WMI: white matter injury. IVH: intraventricular hemorrhage.

© 2019 Benavente-Fernández I et al. *JAMA Network Open.*

eTable 8. Estimated odds ratio (OR) and 95% confidence intervals of the motor outcome associated variables across time through mixed-effects logistic regression.

Number of observations= 520<br>Number of groups (id)= 195<br>Wald chi2(5) = 36.88<br>Log likelihood = -220.29<br>P = 0.0001<br>AUC = 0.95 (95% CI 0.94-0.97).	OR (95% CI)	P
Small for gestational age	5.1 (1.8- 14.8)	0.002
Gestational age	0.8 (0.6- 0.9)	0.007
Severe IVH (grade 3)	12.1 (1.6- 90.1)	0.02
Punctate WMI volume	1.003 (1.001- 1.004)	<0.001
Chronic lung disease	4.8 (1.7- 13.5)	0.003

Adverse motor outcome was considered if M-ABC2 centile rank at 4.5 years ≤15 or if Bayley-III motor score ≤ 85 at 18 and 36 months.

eReferences

1. Callanan C, Doyle L, Rickards A, Kelly E, Ford G, Davis N. Children followed with difficulty: how do they differ? *J Paediatr Child Health.* 2001;37(2):152-156.
2. Hollingshead AB. Four factor index of social status. In: Yale University, NewHa ven, CT; 1975.

© 2019 Benavente-Fernández I et al. *JAMA Network Open.*
